# A Screening Study Identified Decitabine as an Inhibitor of Equid Herpesvirus 4 That Enhances the Innate Antiviral Response

**DOI:** 10.3390/v16050746

**Published:** 2024-05-08

**Authors:** Camille Normand, Côme J. Thieulent, Christine Fortier, Gabrielle Sutton, Catherine Senamaud-Beaufort, Laurent Jourdren, Corinne Blugeon, Pierre-Olivier Vidalain, Stéphane Pronost, Erika S. Hue

**Affiliations:** 1 LABÉO, 14280 Saint-Contest, France; 2 Normandie Université, UNICAEN BIOTARGEN, 14280 Saint-Contest, France; 3 Department of Pathobiological Sciences, School of Veterinary Medicine, Louisiana State University, Baton Rouge, LA 70803, USA; cthieulent@lsu.edu; 4 Louisiana Animal Disease Diagnostic Laboratory, School of Veterinary Medicine, Louisiana State University, Baton Rouge, LA 70803, USA; 5 Normandie Université, UNICAEN, ImpedanCELL, 14280 Saint-Contest, France; 6 Cytokines and Adaptive Immunity Lab, Sainte-Justine University Hospital and Research Center, University of Montréal, Montreal, QC H3T 1C5, Canada; 7 Microbiology, Infectiology and Immunology Department, Faculty of Medicine, University of Montréal, Montreal, QC H3T 1C5, Canada; 8 GenomiqueENS, Institut de Biologie de l’ENS (IBENS), Département de Biologie, École Normale Supérieure, CNRS, INSERM, Université PSL, 75005 Paris, France; 9 Team Viral Infection, Metabolism and Immunity, Centre International de Recherche en Infectiologie (CIRI), Univ Lyon, Institut National de la Santé et de la Recherche Médicale (Inserm), U1111, Centre National de la Recherche Scientifique (CNRS), UMR5308, Ecole Normale Supérieure de Lyon, Université Claude Bernard Lyon 1, 69007 Lyon, France

**Keywords:** real-time cell assay, xCELLigence, antiviral, equid herpesvirus4, EHV-4, decitabine, horse, *Equus caballus*

## Abstract

Equid herpesvirus 4 (EHV-4) is a common respiratory pathogen in horses. It sporadically induces abortion or neonatal death. Although its contribution in neurological disorders is not clearly demonstrated, there is a strong suspicion of its involvement. Despite preventive treatments using vaccines against EHV-1/EHV-4, the resurgence of alpha-EHV infection still constitutes an important threat to the horse industry. Yet very few studies have been conducted on the search for antiviral molecules against EHV-4. A screening of 42 antiviral compounds was performed in vitro on equine fibroblast cells infected with the EHV-4 405/76 reference strain (VR2230). The formation of cytopathic effects was monitored by real-time cell analysis (RTCA), and the viral load was quantified by quantitative PCR. Aciclovir, the most widely used antiviral against alpha-herpesviruses in vivo, does not appear to be effective against EHV-4 in vitro. Potential antiviral activities were confirmed for eight molecules (idoxuridine, vidarabine, pritelivir, cidofovir, valganciclovir, ganciclovir, aphidicolin, and decitabine). Decitabine demonstrates the highest efficacy against EHV-4 in vitro. Transcriptomic analysis revealed the up-regulation of various genes implicated in interferon (IFN) response, suggesting that decitabine triggers the immune antiviral pathway.

## 1. Introduction

Equid herpesvirus 4 (EHV-4, newly designed as *Varicellovirus equidalpha4*) and equid herpesvirus 1 (EHV-1, newly designed as *Varicellovirus equidalpha1*) belong to the Varicellovirus genus classified in the *Alphaherpesvirinae* subfamily (family *Herpesviridae*) [1]. For a long time, they were considered the same virus due to their genetic and antigenic similarities [2,3]. These two viruses, responsible for equine rhinopneumonitis, are enveloped with a linear, double-stranded DNA genome of approximately 145 kbp (EHV-4) and 151 kbp (EHV-1) [4]. The infection caused by EHV-4 mainly affects the upper respiratory tract and is characterized by fever, nasal and/or ocular discharge, and cough in young *Equidae* [5]. In adult and/or vaccinated horses, the infection can be unnoticed or subclinical. EHV-4 also causes sporadic abortions or neonatal death [6]. Unlike EHV-1, the association between the presence of EHV-4 and the nervous form of equine herpesvirus-associated myeloencephalopathy (EHM) has not been clearly demonstrated yet. However, its involvement is highly suspected [7]. EHV-4, like other herpesviruses, can establish lifelong latency in trigeminal ganglia and can be reactivated following stressful conditions (transport, handling, the postpartum period) or after specific treatment (corticosteroid) [8,9]. Studies assessing the prevalence of EHV-4 through serological techniques like ELISAs or Virus Neutralization Tests (VNTs) have reported a rate exceeding 80% [10,11,12,13,14,15]. This corroborates the detection of EHV-4 in trigeminal ganglia by PCR during post-mortem examinations, with rates ranging from 33% to 83% [8,16,17,18,19]. These high values attest to the risk of an EHV-4 epizootic and, thus, the economic risk for the equine industry. Indeed, EHV-4 has a worldwide distribution and causes economic losses due to the cessation of competitions and the need for the establishment of safety precautions during each crisis.

Nowadays, double-valence vaccines against EHV-1 and EHV-4 are available. These vaccines reduce clinical signs and viral excretion [20]. A decrease in the number of abortions has also been described [21]. Despite the vaccination of herds, equine rhinopneumonitis outbreaks due to either EHV-1 or EHV-4 are recorded worldwide [22,23].

To step up the fight against herpesviruses, especially the nervous form associated with EHV-1, research is being carried out into antiviral treatments. As the development of specific antiviral drugs is time-consuming and expensive, «drug repositioning» is being explored as a strategy to fight equine herpesviruses. Aciclovir and ganciclovir, along with their prodrugs (valacyclovir and valganciclovir, respectively), are well characterized for their antiviral activity against human herpesviruses [24]. Consequently, these compounds have been evaluated against EHV-1 both in the field [25,26,27] and during experimental infection [28,29,30], revealing significant benefits in infected horses. As far as EHV-4 is concerned, the therapeutic arsenal is limited and, to our knowledge, only four compounds (aciclovir, ganciclovir, genistein, and dynasore) have been reported to inhibit this virus in vitro [31,32].

We recently screened 2891 molecules against EHV-1 by using real-time cell analysis (RTCA) technology, real-time microscopy, and quantitative PCR (qPCR). This work identified eight molecules capable of inhibiting EHV-1 infection in different cell lines [33,34]. The aim of the present study was (i) to screen the antiviral activity of 42 compounds in an in vitro model of E. Derm cells infected with EHV-4, (ii) to evaluate the effect of the most effective compounds (aphidicolin, cidofovir, decitabine, ganciclovir, idoxuridine, pritelivir, valganciclovir, and vidarabine), and (iii) to study the mode of action of decitabine, identified as the most potent compound against EHV-4.

## 2. Materials and Methods

### 2.1. Cell Line and Virus

Equine dermal fibroblasts (E. Derm, NBL-6 CCL-57™; ATCC^®^, Manassas, VA, USA) were maintained in Eagle’s Minimum Essential Medium (EMEM; ATCC^®^, Manassas, VA, USA) supplemented with 10% fetal bovine serum (FBS) (Eurobio, Courtaboeuf, France), 100 UI/mL penicillin, 0.1 mg/mL streptomycin, and 0.25 µg/mL amphotericin B (Eurobio) and cultivated at 37 °C and 5% CO_2_. Cells were seeded at 1.2 × 10^4^ cells/well in 96-well plates.

The EHV-4 405/76 strain (VR-2230™; ATCC^®^) was used as the reference strain for the screening of the antiviral effect of compounds at an MOI of 0.23 on E. Derm cells.

### 2.2. Compounds

This study included 40 compounds (herein called an in-house antiviral library) previously evaluated in Thieulent et al.’s 2020 study; they were selected for their effects against different human viruses [33]. Two other molecules (genistein and dynasore) were selected due to their antiviral effect against EHV-4 in vitro, as shown by Spiesschart et al. [31]. All compounds were dissolved in dimethyl sulfoxide (DMSO, Sigma-Aldrich, Saint Quentin Fallavier, France) to prepare 10 or 20 mM stock solutions (Supplementary Table S1). These solutions were aliquoted and stored at −20 °C until use.

### 2.3. Screening of Antiviral Effect by Real-Time Cellular Analysis

Screening of the 42 compounds was carried out at four concentrations (0.4, 2, 10, and 50 μM) with EHV-4-infected cells by impedancemetry using the RTCA MP system (xCELLigence^®^; ACEA Biosciences Inc., San Diego, CA, USA) as described previously [33]. Firstly, 50 µL of medium was added to the wells to perform background impedance readings. Next, 100 µL of cells was seeded in an E-plate 96 at the density cited in Section 2.1, and the cells were incubated at room temperature for 30 min before being incubated in the device station at 37 °C and 5% CO_2_. After 24 h of incubation, the medium was removed, and the cells were infected and treated with 5-fold serial dilutions of each compound. The plates were put back onto the station at 37 °C and 5% CO_2_ for 120 h post-infection (hpi). The control cells were treated with 0.5% of DMSO in the presence or absence of the virus. The same pipeline used by Thieulent et al. was applied to analyze data [32,33]. First, the normalized area under the curve (AUC_n_) from 0 to 120 hpi was calculated for the control wells. The results from a screening plate were considered valid when the Z’-factor calculated from the AUC values of the control wells was above 0.5 [35]. Next, the AUC_n_ was calculated for each compound, as well as the time required for the Cell Index (CI) to decrease by 50% (CIT_50_) after viral infection. A compound was considered to have an antiviral effect when (i) the AUC_n_ increased by >25% and (ii) the CIT_50_ was delayed by >8 h compared to the DMSO-treated infected cells.

**Figure 1:**
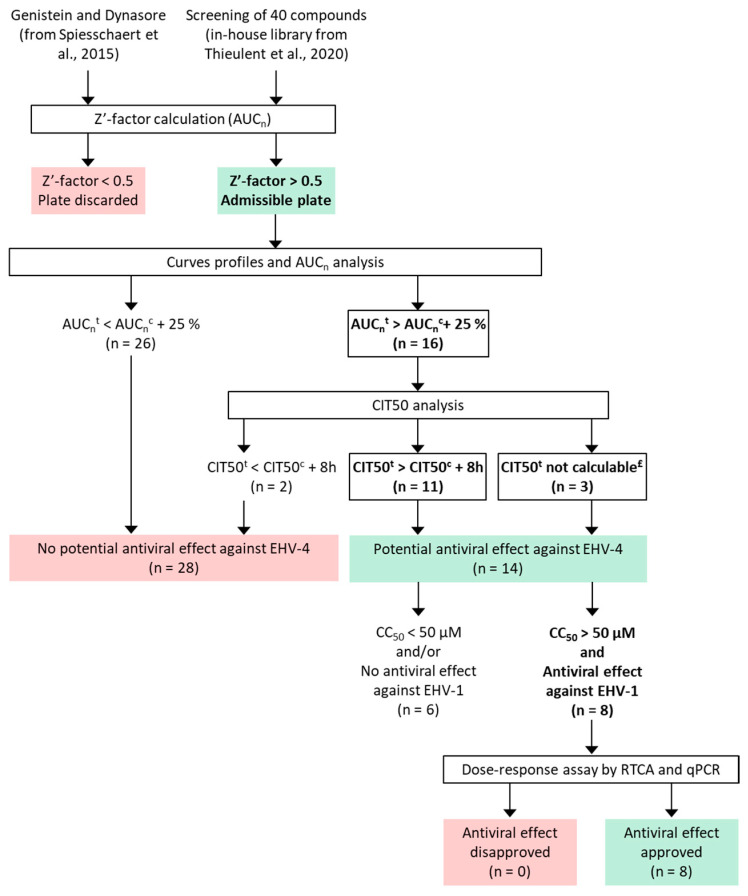
Decision tree for the analysis of data from the screening of 42 compounds ([31,33]) in a 96-well format using (1) RTCA screening and (2) dose response assay by RTCA and qPCR. AUC_n_ corresponds to the area under normalized curves. AUC_n_^t^ is the area under normalized curves of the treated cells. AUC_n_^c^ is the area under normalized curves of the DMSO-treated control cells. CIT_50_^t^ is the time required for the CI_n_ of the treated cells to decrease by 50% after virus infection, and CIT_50_^c^ is the time required for the CI_n_ of the DMSO-treated control cells to decrease by 50% after virus infection. ^£^ CIT_50_^t^ not calculable: treatments prevent the CI_n_ decrease. CC_50_ refers to the half-maximal cytotoxic concentration. The criteria established for the screening step and the determination of EC_50_ values step are indicated in bold.

Among the 14 compounds satisfying the above criteria, 8 drugs were chosen according to additional criteria corresponding to (i) the absence of toxicity on E. Derm cells at all concentrations tested (half-maximal cytotoxic concentration [CC_50_] > 50 µM) in Thieulent et al.’s 2020 [33] and (ii) an antiviral effect against EHV-1 in our previous report [33] (Figure 1). The antiviral effects of cidofovir (CDV), ganciclovir (GCV), idoxuridine (IDU), pritelivir (BAY 57-1293), valganciclovir (VGCV), and vidarabine (VDR) were tested from 50 to 0.39 µM by 2-fold serial dilution. The concentrations used for decitabine (DTB) ranged from 50 to 0.10 µM. Finally, the antiviral activity of aphidicolin (APD) was evaluated for concentrations from 10 to 0.08 µM. Then, the calculation of the percentage of virus inhibition was performed, and dose–response curves were obtained. The percentage of viral inhibition was estimated through the adapted formula described by Pan et al. (2013) [36]:

$$\mathrm{viral}\;\mathrm{inhibition}\;(\%)=100\times (1-\frac{\left(a-b\right)}{\left(c-b\right)})$$

where *a* corresponds to the AUC_n_ of infected cells treated with different concentrations of compounds, *b* indicates the AUC_n_ of control cells treated with 0.5% of DMSO, and *c* is the AUC_n_ of EHV-4-infected cells treated with 0.5% of DMSO.

### 2.4. Viral Quantitation by qPCR Assay

Cells were seeded in 96-well plates, infected with the EHV-4 405/76 strain, and treated or not as described in part 2.3. At 48 hpi, the plates were frozen at −80 °C. After one freeze/thaw cycle, nucleic acids were extracted using the NucleoMag Pathogen Kit (Macherey-Nagel, Hoerdt, France) and placed onto the KingFisher Flex Purification System (Thermofisher, Courtaboeuf, France). The nucleic acids were stored at −20 °C until use. Each thermal cycling was performed on a QuantStudio™ 12 K Flex Real-Time PCR System (Life Technologies, Courtaboeuf, France). The quantitative PCR (qPCR) protocols for EHV-4 were performed as previously described [32,37]. The quantification of viral genome copies was used to determine the percentage of viral inhibition as described in Section 2.3.

### 2.5. Transcriptomic Analysis

#### 2.5.1. Experiments

E. Derm cells were seeded at 2.5 × 10^4^ cells/well in 6-well plates (Falcon, Falmouth, UK) and incubated at 37 °C and 5% CO_2_. Twenty-four hours post-seeding, the cells were infected with the EHV-4 405/76 strain at an MOI = 2 and treated or not with 25 µM of DTB. At 18 hpi, the medium was removed, and the cells were rinsed two times with antibiotics- and FBS-depleted medium. Then, the cells were lysed with 350 µL of RLT buffer complemented with β-mercaptoethanol and frozen at −80 °C. After one freeze/thaw cycle, nucleic acids were extracted using the RNeasy Plus mini kit (Qiagen, Courtaboeuf, France) according to the manufacturer’s instructions and stored at −80 °C.

#### 2.5.2. Libraries

Library preparation and Illumina sequencing were performed at the Ecole Normale Supérieure Genomique, ENS core facility (Paris, France). Messenger (polyA+) RNAs were purified from 200 ng of total RNA using oligo(dT). Libraries were performed using the strand-specific RNA-Seq library preparation Stranded mRNA Prep Ligation kit (Illumina, San Diego, CA, USA) and were multiplexed by 26 to 28 on 5 P3 flowcells and on additional a P2 flowcell with 6 samples (Illumina). A 68 bp single-end read sequencing was performed on a NextSeq 2000 device (Illumina). A mean of 41 ± 12 million passing Illumina quality filter reads was obtained for each of the 12 samples.

#### 2.5.3. RNASeq Bioinformatics Analysis

The analyses were performed using the Eoulsan pipeline [38], including read filtering, mapping, alignment filtering, read quantification, normalization, and differential analysis: Before mapping, poly N read tails were trimmed, reads ≤ 40 bases were removed, and reads with a mean quality ≤ 30 were discarded. The reads were then aligned against the *Equus caballus* genome from Ensembl version 108 and viral genomes from NCBI (KT324740 for EHV-4) using STAR (version 2.78a) [39]. Alignments from reads matching more than once on the reference genome were removed using the Java version of samtools [40]. To compute gene expression, *Equus caballus* GTF genome annotation version 108 from Ensembl database enhanced with EHV-4 virus annotations from NCBI was used. All overlapping regions between alignments and referenced exons were counted and aggregated by genes using HTSeq-count 0.5.3 [41]. The sample counts were normalized using DESeq2 1.8.1 [42]. Statistical treatments and differential analyses were also performed using DESeq2 1.8.1. The RNASeq gene expression data and raw fastq files are available in the GEO repository (www.ncbi.nlm.nih.gov/geo/, e.g., accessed on 19 March 2024) under accession number: GSE261894.

The enrichment analysis of the differentially expressed genes (DEGs) was performed with the DAVID (Database for Annotation, Visualization and Integrated Discovery) database for Gene Ontology (GO) with Knowledgebase v2023q4 [43,44]. DEGS were chosen with |Log2(FC)| > 1 and adjusted *p*-value < 0.05 with Benjamini–Hochberg correction. The GO was divided into biological processes (BPs), molecular functions (MFs), and cellular components (CCs) analyses. DEGs considered as significantly enriched passed a cut-off value of an adjusted *p*-value < 0.05. A radar chart of the 11 up-regulated genes involved in the “response to virus” GO term was made with the fmsb package available in the CRAN repository using RStudio version 4.3.2.

### 2.6. Statistical Analysis

EC_50_ and CC_50_ values were determined using a non-linear regression dose response inhibition curve (GraphPad Prism^®^ software 10.1.2; La Jolla, CA, USA). The Selectivity Index (SI) was calculated for each compound using the following formula:

$$\mathrm{SI}=\frac{{CC}_{50}}{{EC}_{50}}$$

## 3. Results 

### 3.1. Screening and Selection of the Most Effective Compounds against EHV-4

To identify novel antiviral compounds against EHV-4, our in-house antiviral library (40 molecules) and 2 molecules reported by Spiesschaert et al. were screened in 96-well plates at four concentrations (0.4, 2, 10, and 50 μM) by an impedance-based (RTCA) system using E. Derm cells infected with the EHV-4 405/76 reference strain [31]. To ensure the robustness of our assay, the Z′ factors of the six screening plates were calculated based on AUC_n_ values calculated from the RTCA curves of the control wells. Values between 0.59 and 0.92 were obtained, with a median of 0.77, thus validating the assay. The potential antiviral activity of the tested compounds was determined based on AUC_n_ and CIT_50_ values calculated from the RTCA curves of the treated wells, as described in Section 2.3. Based on applied criteria, 14 of the 42 compounds displayed potent antiviral activity against EHV-4: adefovir dipivoxil, aphidicolin (APD), BAY 57-1293, cidofovir (CDV), cytarabine, decitabine (DTB), favipiravir, ganciclovir (GCV), idoxuridine (IDX), maribavir, proguanil hydrochloride, tenofovir disoproxil, valganciclovir hydrochloride (VGCV), and vidarabine (VDR) (Table 1). Aciclovir, genistein, and dynasore did not show antiviral activity in this experimental setting. 

### 3.2. Evaluation of the Antiviral Effect of Eight Antiviral Drugs of Interest against EHV-4

We selected a subset of eight molecules meeting additional criteria: (i) the absence of toxicity on E. Derm cells at all tested concentrations ([CC_50_] > 50 µM) and (ii) an antiviral effect against EHV-1 in our previous study [33], therefore allowing for the identification of compounds inhibiting both EHV-1 and EHV-4 to prevent equine rhinopneumonitis. This short list included two acyclic guanosine analogs (GCV and VGCV), one acyclic guanosine analog (CDV), one acyclic adenosine analog (VDR), and one deoxyuridine analog (IDX). All these five compounds are known inhibitors of herpesviruses’ DNA polymerase [24]. Decitabine (DTB), a deoxycytidine analog altering cellular DNA methylation used for Acute Myeloid Leukemia treatment, was also selected [45]. Finally, aphidicolin (APD), a tetracyclic diterpene antibiotic, and pritelivir (BAY 57-1293), an inhibitor of the Herpes simplex virus-1 (HSV-1) helicase–primase complex [46], were included. All eight molecules were chosen for further evaluation against EHV-4 in dose–response experiments. 

The in vitro efficacy of the eight compounds was evaluated by both RTCA and qPCR methods. The EC_50_ value of each compound was calculated from the percentage of viral inhibition determined with AUC_n_ values or the quantification of viral genome copies, respectively. The results are presented in Table 2. 

IDX presented weak antiviral activity against EHV-4, with an EC_50_ value of 33.75 ± 15.85 µM by RTCA and 7.49 ± 1.00 µM by qPCR. CDV, BAY 57-1293, and VDR showed intermediate antiviral activity against EHV-4 (from 12.55 ± 4.32 to 17.77 ± 1.20 µM by RTCA). Similarly, the EC_50_ values of VGCV were 9.55 ± 2.81 µM by RTCA and 3.60 ± 1.28 µM by qPCR. GCV had stronger antiviral activity against EHV-4, with EC_50_ values measured by the RTCA system and qPCR of 4.05 ± 0.22 µM and 1.32 ± 1.06 µM, respectively.

**Figure 2:**
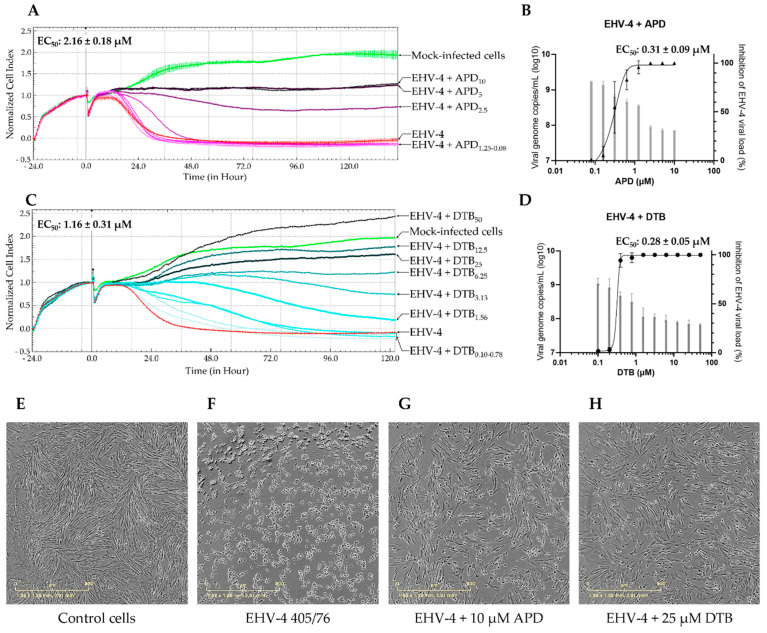
Aphidicolin and decitabine inhibit EHV–4 405/76–induced cytopathic effect and viral replication, as determined by RTCA and qPCR. Real-time monitoring by impedancemetry of cytopathic effect induced by EHV-4 in the presence of aphidicolin (APD) (**A**) or decitabine (DTB) (**C**). The black vertical line corresponds to the normalization time, which is the last time point before infection. The green curve represents the normalized Cell Index (CI_n_) of the mock-infected cells. The red curve represents the CI_n_ of the untreated EHV–4–infected cells. The purple- and blue-shaded curves indicate the CI_n_ of the EHV–4–infected cells treated with a two–fold serial dilution of APD or DTB, respectively. Also shown is the viral genome copy number of EHV–4 measured by qPCR at 48 hpi after treatment with eight concentrations of APD (**B**) or DTB (**D**). Bars correspond to viral load, whereas dots represent the percentage of inhibition. The EC_50_ values were determined from the percentage of the viral load. Each point represents the mean ± SD of three independent experiments. Microscopic observation of mock-infected cells (**E**) or cells infected with EHV–4 405/76 reference strain (**F**) after a treatment with 10 µM of APD (**G**) or 25 µM of DTB (**H**) realized at 48 hpi.

APD and DTB were the most potent compounds identified against EHV-4 in this study. The CIT_50_ increased by 22.43 ± 4.89 h and 7.48 ± 2.69 h at 2.5 and 1.25 µM of APD, respectively (Figure 2A). APD had EC_50_ values of 2.16 ± 0.18 µM, as determined in the RTCA system (Figure 2A). An EC_50_ value of 0.31 ± 0.09 µM was estimated by qPCR (Figure 2B). In the presence of 1.56 µM and 0.78 µM of DTB, the CIT_50_ values increased by 30.13 ± 15.34 and 25.66 ± 10.74 h, respectively (Figure 2C). Next, the EC_50_ values of DTB were determined by RTCA and qPCR. DTB presented an EC_50_ value of 1.16 ± 0.31 µM by RTCA assay (Figure 2C) and an EC_50_ value of 0.28 ± 0.05 µM by qPCR assay (Figure 2D). All these results agree with the cellular morphology observed by microscopy (Figure 2E–H). Altogether, these results show that DTB is the most interesting compound among the eight molecules tested. This led us to further investigate its mode of action to explain the antiviral effect.

### 3.3. Transcriptomic Analysis of DTB-Treated Cells

**Figure 3:**
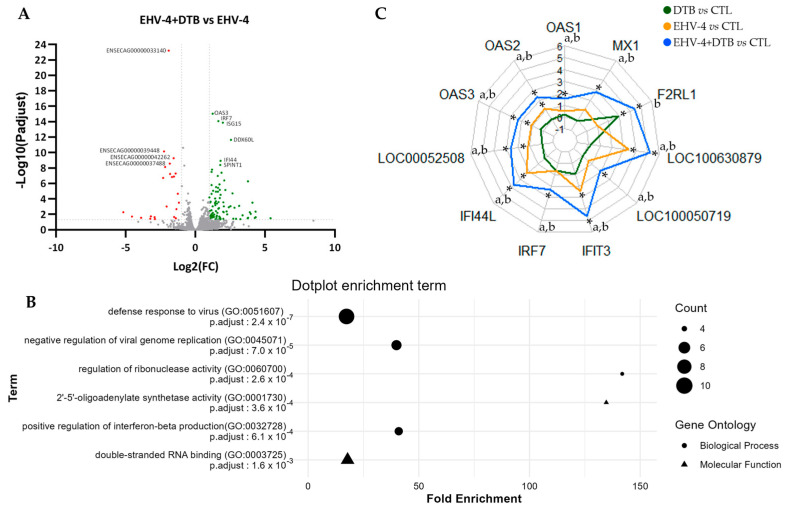
Effect of DTB on the transcriptome of E. Derm cells infected with EHV–4 405/76. (**A**) Volcano plot showing DEGs in EHV–4–infected cells treated with DTB (EHV–4+DTB) when compared to non–treated EHV–4–infected cells (EHV–4). The *x*–axis represents the Log2 fold changes with a cutoff of |Log2(FC)| > 1. The y–axis indicates the –Log10 of the adjusted *p*-values (with Benjamini–Hochberg correction) with a cutoff *p*-value adjusted < 0.05. Gray dots are the non-significant DEGs, red dots represent the significant down-regulated DEGs, and green dots correspond to the significant up-regulated DEGs. The DEGs with the highest adjusted *p*-value are annotated. (**B**) Gene Ontology (GO) enrichment analysis of DEGs. Results of the six significant GO terms showing four biological processes (circle) and two molecular functions (triangle). The size of the dots represents the number of genes. (**C**) Radar chart showing the differential expression of the 11 DEGs implicated in the «defense response to virus» GO term. The axis represents the Log2 fold change for DTB vs. CTLs, EHV–4 vs. CTLs, or EHV–4+DTB vs. CTLs. a represents the significantly different expression levels for EHV–4+DTB vs. DTB. b indicates a significant level of expression of the DEGs for EHV–4+DTB vs. EHV–4. * shows the significant differences from the CTLs with an adjusted *p*-value < 0.05 with Benjamini–Hochberg correction.

To better understand the antiviral effect of DTB, we compared the transcriptome of the EHV-4-infected cells in the absence or presence of DTB. Our comparison of the EHV-4+DTB and EHV-4 conditions identified 119 DEGs, including 94 up-regulated and 25 down-regulated DEGs (Figure 3A). We then looked for enriched functional annotations in the list of up-regulated genes using the Gene Ontology (GO) database. The results of the GO enrichment analysis revealed that the DEGs are significantly (FDR < 0.05) involved in four biological processes (BP): the “defense response to virus”, “negative regulation of viral genome replication”, “regulation of ribonuclease activity”, and “positive regulation of interferon-beta production”. Interestingly, three genes are shared by these four biological processes: ENSECAG00000013435 (OAS1), ENSECAG00000014422 (OAS2), and ENSECAG00000008809 (OAS3). Accordingly, the list of DEGs was also enriched for two molecular functions (MFs): “2′-5′-oligoadenylate synthetase activity” and “double-strand RNA binding” (Figure 3B). We thus analyzed the expression of the 11 DEGs associated with the GO term “defense response to virus” using transcriptomic data from E. Derm cells that were either untreated (CTLs), treated with DTB alone (DTB), infected with EHV-4 (EHV-4), or infected with EHV-4 and treated with DTB (EHV-4+DTB). As shown in Figure 3C, DTB alone only induced F2RL1 but showed no effect on the expression of the other genes. Infection with EHV-4 alone induced about half of the genes (6 out of 11). However, when EHV-4-infected cells were treated with DTB, all eleven genes were induced, and the expression of those induced by EHV-4 alone was further increased. Overall, this shows that DTB stimulates the expression of antiviral genes in EHV-4-infected cells.

## 4. Discussion

Respiratory infections induced by equine herpesvirus are endemic worldwide and lead to economic and animal welfare losses for the equine industry. EHV-1 and EHV-4 are the main agents responsible for respiratory infections in horses, with varying degrees of intensity. These viruses also cause abortion in the third trimester of gestation or neonatal death. In order to maximize the disease’s treatment and welfare grounds, antiviral molecules were used in the field on horses [25,26,27]. In experimental infections, valacyclovir (the pro-drug of aciclovir) has shown mixed results on clinical and virological parameters [28,29]. Also, experimental infections with EHV-1 for testing different treatments remain limited due to ethical considerations regarding the secondary forms of the disease, costs, and animal welfare. Unlike EHV-1, EHV-4 infection is usually limited to the respiratory form of the disease, and its involvement in the nervous form has not been demonstrated. Therefore, EHV-4 could serve as a promising alternative for the initial in vivo evaluation of experimental treatments against equine herpesviruses. In addition, it would be very convenient to have antiviral molecules that are active on these two herpesviruses, irrespective of viral species or strain. These reasons motivated the current work and our specific focus on molecules inhibiting both EHV-1 and EHV-4. We started with a subset of 40 compounds, selected for their effects against different human viruses, plus 2 molecules reported for their antiviral activity against EHV-4 [31,33]. The screening against EHV-4 was conducted by impedancemetry and qPCR as previously described [32,33,34]. Among the 14 molecules identified, only 8 (aphidicolin, cidofovir, decitabine, ganciclovir, idoxuridine, pritelivir (BAY 57-1293), valganciclovir, and vidarabine) were selected for inclusion in this study. A limitation of this repositioning strategy is that it limits the discovery of new molecules with efficacy against EHV-4 only, but our goal was to identify compounds with a potent efficacy against EHV-1 and EHV-4. The discovery of new compounds, even by repositioning, is a long and costly process, and with this strategy, we hope to identify compounds with a broad spectrum of applications in the equid alpha-herpesvirus family.

Aciclovir is the only antiviral compound used in the field by veterinarian practitioners to fight EHV-1. In vitro and in vivo studies have demonstrated that aciclovir lacks antiviral activity against EHV-1 [28,32]. Our study confirms the inefficiency of aciclovir against EHV-4, which was already highlighted by two previous works [32,47]. This could explain the lack of efficacy often observed in the field and increase the interest in finding new compounds with a better efficacy. Alongside aciclovir, our screening also identified 28 other compounds with no antiviral effect against EHV-4. Among them, we found statin-based molecules and the 25-hydroxycholesterol. These results are quite surprising due to their antiviral effect detected in repositioning against human and veterinarian viruses [48,49,50]. Dynasore and genistein did not present any antiviral effect, which is in discordance with the study of Spiesschaert et al. [31]. This difference may be due to differences in treatment protocol. In the study reported by Spiesschaert et al., these compounds were used at 310 and 370 µM, respectively, and the cells were pre-treated for 1 h prior to infection. In our study, the concentration of the molecules was 50 µM, and the treatment took place at the same time as the addition of the compound. 

The antiviral effect detected during the screening was then further evaluated by a dose–response assay for a subset of eight compounds. Antiviral activity was confirmed for all of them. 

Idoxuridine was the first anti-herpesvirus drug approved by the FDA in 1962 for topical treatment for keratitis caused by the HSV. Idoxuridine is still used against HSV-1 and HSV-2, but it is used less frequently due to its toxicity [24]. Vidarabine was the first molecule to be used as a systemic treatment for human herpesviruses. Once again, its use is limited because of the disadvantages (toxicity) of the treatment and the discovery of more potent antiviral drugs [24]. 

Pritelivir is an inhibitor of the HSV-1 helicase–primase complex first described by Kleymann et al. in 2002 [51]. Due to its different mode of action, BAY 57-1293 was developed to counter aciclovir-resistant strains of the HSV. To our knowledge, since this study, only new data obtained by our team have been recorded on EHV-1 with the repositioning approach [33]. Similar data were observed in this study on EHV-4. However, these data differed from the results obtained for the HSV [52]. 

The antiviral effect of cidofovir, a nucleotide analog, has been tested on a wide range of viruses, and EC_50_ values vary between different virus families and strains. Regarding equid herpesviruses, the EC_50_ of cidofovir determined on EHV-4 in this study is consistent with previous studies on EHV-1 and EHV-3 [33,53]. 

Ganciclovir and its prodrug, valganciclovir, are two compounds approved for use in human medicine to treat herpesviruses such as the HSV and human cytomegalovirus (HCMV). Following the first study reporting the antiviral effect of ganciclovir against equid herpesviruses 1 and 3 [54], which was reported in 1983, in 2007, Garré et al. published a work presenting EC_50_ values between 0.1 and 0.4 µg/mL against EHV-1 [55]. The pharmacokinetics of ganciclovir (administered intravenously) and valganciclovir (administered orally) were evaluated in horses [56]. The intravenous administration of a dose of 2.5 mg/kg every 8 h for 24 h followed by doses of 2.5 mg/kg every 12 h kept the concentration of ganciclovir in horse sera higher than the EC_50_ value determined in vitro. This study also demonstrates that the oral administration of valganciclovir improves the bioavailability of ganciclovir by 41 ± 20%. Other studies performed in vitro to prevent EHV-1- and EHV-3-mediated infection demonstrate the interest in this molecule [32,33,53]. In the present study, performed in vitro with EHV-4, the EC_50_ values of ganciclovir and valganciclovir were found to be 4.05 µM and 9.55 µM by RTCA and 1.32 µM and 3.60 µM by qPCR, respectively. These results are in agreement with those published previously by Azab et al. and Thieulent et al. [32,47]. 

In 2016, RTCA technology was used by Piret et al. to study the efficacy of ganciclovir and foscarnet drugs against HCMV [57]. The efficacy of ganciclovir against EHV-4 in vitro is in line with that against HCMV by RTCA. More recently, treatment with valganciclovir was tested against EHV-1-infected ponies, and the results showed that the drug reduced the shedding and the viraemia in the infected group compared with the control group [30]. Ganciclovir was used to prevent infection in other species like feline herpesvirus 1 (FHV-1) and canine herpesvirus 1 (CHV-1). Although data against FHV-1 are consistent with our study, those obtained against CHV-1 are different, but again, this result could be explained by the differences in the methodologies of the experiments [58].

In 2007, Goodman et al. studied the efficacy of aphidicolin against EHV-1. Their study indicates that two strains of EHV-1 are susceptible to aphidicolin [59]. In previous studies, aphidicolin also showed potent antiviral activity against EHV-1 [33,60]. In high concentrations, aphidicolin has been shown to have a cytostatic effect on cells. The antiviral effect observed for aphidicolin against EHV-4 could therefore be due to an effect via the cell cycle rather than on virus replication. This hypothesis could be explained by the compound’s mode of action, being an inhibitor of DNA polymerase alpha, as suggested by Sheaff et al. [61].

Decitabine (DTB) has been identified as the most effective compound against EHV-4 in vitro. This compound is a deoxycytidine analog and hypomethylating agent used in the treatment of myelodysplastic syndromes [45,62]. In mice, the toxic concentration of decitabine was estimated to be 25 µM [63]. DTB was shown to modify the methylation of the Epstein–Barr virus genome [64], and our team documented its antiviral activity by showing an inhibitory effect on EHV-1 replication [33]. The current study shows that DTB has antiviral activity against EHV-4, reinforcing its potential for treating rhinopneumonitis. Thieulent et al. proposed that DTB, as a nucleoside analog, is incorporated into the DNA genome of EHV-1 during viral replication and/or impedes the viral polymerase activity, which leads to the inhibition of viral growth [33]. Our transcriptomic analysis identified 119 DEGs that were either up- or down-regulated upon DTB treatment in EHV-4-infected cells. An analysis of the GO terms performed on the subset of 94 up-regulated genes revealed a significant enrichment of the GO terms related to viral replication, in particular 11 genes associated with the “defense response to virus” (GO:0051607). These genes correspond to OAS1, OAS2, OAS3, 2′-5′-oligoadenylate synthase-like protein 2, IFI44L, IRF7, IFIT3, interferon-induced transmembrane protein 3 (IFITM3), interferon-induced protein with tetratricopeptide repeats 1B (IFIT1B), F2RL1, and MX1, which are all implicated in the interferon response. Although DTB was unable to induce these genes on its own, with the exception of F2RL1, it amplified their expression upon infection with EHV-4. It seems that DTB helps to restore the innate immune response of cells infected with this virus. This result is in accordance with what has been previously shown. Indeed, DTB is already known as a stimulator of the IFN pathway and the expression of interferon-stimulated genes (ISGs). In particular, it has been shown that DNA methyltransferase inhibitors such as DTB reactivate the expression of endogenous retroviruses (ERVs), leading to the production of double-stranded RNA molecules that are capable of inducing interferons and ISGs through the TLR3 or the MAVS pathways [65,66]. Additionally, four of the eleven genes whose expression is enhanced by DTB are oligoadenylate synthetases or OASs. These enzymes are activated by dsRNA molecules to produce 2′-5′ oligoadenylates (2-5A) that act as a second messenger to activate the latent ribonuclease or RNAseL. This OAS/RNAseL system is a potent IFN effector pathway, and it has been suggested that herpes simplex virus escapes this antiviral mechanism by stimulating the production of inactive forms of 2-5A [67]. The potent antiviral effect of DTB could be explained by two mechanisms. First, DTB treatment could demethylate cellular DNA, thus inducing the expression of genes involved in the innate antiviral response, as previously described. Secondly, DTB could act as a cytidine analog inhibiting viral DNA replication and exerting a direct antiviral effect.

Although antiviral effects were observed in our study, it is necessary to confirm the antiviral activities of these compounds by ex vivo experiments using organoid models or before in vivo experiments. For example, aciclovir presents an antiviral effect in vitro on cell lines infected with EHV-1 [33,47,55] but does not prevent the infection of equine nasal respiratory explants in an ex vivo model [68]. In addition, the experimental infection of ponies with EHV-1 and treated with valaciclovir did not show any effect of the molecule with respect to reducing clinical signs, nasal shedding, or viremia [28]. Today, organoids are already used in human medicine to study the pathogenesis of a virus and to evaluate the impact of using an antiviral molecule. In 2022, Rybak-Wolf et al. published an article about viral encephalitis induced by HSV-1 in cerebral organoids and concluded that aciclovir treatment stopped viral replication but did not prevent HSV-1-driven defects [69]. Furthermore, the development of organoids in veterinary medicine seems to be already in progress and could be a major advance in research on the antiviral properties of compounds [70,71,72]. 

## 5. Conclusions

In conclusion, the results presented here confirm the ability of ganciclovir to inhibit the replication of EHV-4 in E. Derm cells. No antiviral activity of aciclovir was detected, and the two previously tested compounds, dynasore and genistein, failed to inhibit EHV-4 replication in vitro. Aphidicolin, cidofovir, pritelivir, valganciclovir, and vidarabine present antiviral activity and warrant further investigations. DTB is the most potent compound identified to inhibit EHV-4 replication in vitro. The transcriptomic analysis of DTB-treated infected cells revealed an activation of the innate antiviral response by stimulating the IFN pathway. The combination of DTB with other previously identified compounds requires further investigation in vitro. 

## Figures and Tables

**Table 1:** Results of the screening of the antiviral activities of the 42 compounds against EHV-4 405/76 in vitro by RTCA. The most important resultats are bolding. AUC_n_: area under normalized Cell Index curves, %AUC_n_: AUC_n_ of treated EHV-4-infected cells compared to AUC_n_ of untreated EHV-4-infected control cells, CIT_50_: time required for the Cell Index to decrease by 50% after virus infection, ∆CIT_50_: difference between the CIT_50_ of the treated EHV-4-infected well and the CIT_50_ of the untreated EHV-4-infected control well, N.T.: not tested, N.D.: not determinable.

		EHV-4															
Compound Name	Z’factor	AUC_n_				%AUC_n_				CIT_50_				ΔCIT_50_			
		50 µM	10 µM	2 µM	0.4 µM	50 µM	10 µM	2 µM	0.4 µM	50 µM	10 µM	2 µM	0.4 µM	50 µM	10 µM	2 µM	0.4 µM
2′-C-methylcytidine	0.59	30.32	26.93	35.13	37.52	−24.38	−32.85	−12.40	−6.43	33:13:54	33:29:59	40:03:25	41:23:10	00:00:00	00:00:00	00:00:00	00:00:00
25-hydroxycholesterol	0.59	31.86	29.79	34.81	39.29	−20.54	−25.72	−13.19	−2.03	50:17:48	33:54:28	41:47:24	41:04:43	6:28:32	00:00:00	00:00:00	00:00:00
Abacavir sulfate	0.77	32.99	31.76	32.21	38.16	−7.59	−11.03	−9.79	6.89	30:53:42	33:52:45	32:41:22	39:39:32	00:00:00	00:00:00	00:00:00	3:20:47
Aciclovir	0.84	55.9	29.1	33.7	36.7	40.30	−26.91	−15.45	−7.83	25:08:54	23:14:22	19:57:36	20:34:14	00:00:00	00:00:00	00:00:00	00:00:00
Adefovir dipivoxil	0.77	39.70	73.47	93.75	99.92	11.19	105.80	162.61	179.88	N.D.	83:42:08	N.D.	N.D.	N.D.	47:23:23	N.D.	N.D.
**Aphidicolin**	**0.84**	**N.T.**	**106.29**	**81.72**	**27.44**	**N.T.**	**166.79**	**105.13**	**−31.13**	**N.T.**	**N.D.**	**31:52:38**	**18:12:03**	**N.T.**	**N.D.**	**01:56:34**	**00:00:00**
Arbidol	0.59	35.06	37.59	39.33	31.88	−12.56	−6.26	−1.93	−20.51	44:30:54	42:10:11	42:41:50	33:59:50	0:41:38	00:00:00	00:00:00	00:00:00
Atorvastatin	0.59	13.93	17.36	27.80	40.71	−65.25	−56.70	−30.67	1.53	14:49:40	16:02:38	31:27:28	49:00:07	00:00:00	00:00:00	00:00:00	5:10:51
**BAY 57-1293**	**0.92**	**145.59**	**44.70**	**27.62**	**27.43**	**318.36**	**28.45**	**−20.63**	**−21.19**	**N.D.**	**46:14:40**	**30:05:06**	**30:36:01**	**N.D.**	**9:43:03**	**00:00:00**	**00:00:00**
Brivudine	0.92	36.82	31.74	30.37	28.13	5.81	−8.78	−12.72	−19.17	36:00:28	33:58:03	30:48:06	33:15:21	00:00:00	00:00:00	00:00:00	00:00:00
Capecitabine	0.77	33.97	36.13	36.42	35.92	−4.85	1.21	2.01	0.61	34:05:31	34:59:06	36:11:11	37:49:04	00:00:00	00:00:00	00:00:00	1:30:19
**Cidofovir**	**0.59**	**59.40**	**40.56**	**42.07**	**38.54**	**48.13**	**1.14**	**4.91**	**−3.89**	**65:56:11**	**52:15:11**	**44:56:43**	**43:26:35**	**22:06:55**	**8:25:55**	**1:07:27**	**00:00:00**
Cytarabine	0.77	91.89	91.79	32.46	28.59	157.38	157.12	−9.07	−19.93	N.D.	N.D.	34:30:53	30:34:51	N.D.	N.D.	00:00:00	00:00:00
**Decitabine**	**0.77**	**128.21**	**120.54**	**93.80**	**73.51**	**259.12**	**237.65**	**162.75**	**105.91**	**N.D.**	**N.D.**	**65:52:36**	**61:28:17**	**N.D.**	**N.D.**	**29:33:51**	**25:09:32**
Didanosine	0.77	38.91	29.27	36.23	37.77	8.99	−18.02	1.50	5.81	37:24:28	34:16:07	37:23:48	42:13:43	1:05:43	00:00:00	1:05:03	5:54:58
DMXAA	0.59	60.10	41.23	37.99	36.50	49.87	2.81	−5.27	−8.97	50:24:42	38:56:11	41:35:59	39:08:28	6:35:26	00:00:00	00:00:00	00:00:00
Dynasore	0.76	23.18	18.85	13.81	13.10	63.56	33.01	−2.53	−7.53	20:21:09	19:38:53	15:26:11	15:01:12	4:31:40	03:49:24	00:00:00	00:00:00
Eflornithin (dfmo)	0.59	32.46	30.27	32.10	44.63	−19.06	−24.51	−19.95	11.31	35:16:47	34:06:42	36:27:35	49:20:45	00:00:00	00:00:00	00:00:00	5:31:29
Emtricitabine	0.77	37.28	34.12	35.47	36.81	4.43	−4.43	−0.64	3.10	34:46:56	35:10:59	34:12:33	38:55:58	00:00:00	00:00:00	00:00:00	2:37:13
Famciclovir	0.77	37.57	36.03	34.29	39.12	5.24	0.93	−3.95	9.57	39:14:00	36:41:25	33:42:43	37:03:11	2:55:15	0:22:40	00:00:00	0:44:26
Favipiravir	0.59	74.44	37.23	41.18	30.35	85.63	−7.16	2.69	−24.32	56:41:52	41:21:34	42:08:47	32:01:19	12:52:36	00:00:00	00:00:00	00:00:00
5-fluorouracil	0.59	36.75	31.13	31.41	39.89	−8.36	−22.38	−21.67	−0.53	32:01:21	32:46:38	35:53:16	49:10:00	00:00:00	00:00:00	00:00:00	5:20:44
Fluvastatin	0.59	13.86	11.66	16.45	33.08	−65.44	−70.91	−58.97	−17.51	15:02:49	13:46:46	15:06:58	41:48:06	00:00:00	00:00:00	00:00:00	00:00:00
**Ganciclovir**	**0.84**	**138.7**	**116.3**	**74.3**	**57.9**	**248.20**	**191.80**	**86.60**	**45.30**	**N.D.**	**N.D.**	**19:01:38**	**18:32:55**	**N.D.**	**N.D.**	**00:00:00**	**00:00:00**
Genistein	0.76	11.8	13.3	12.8	11.7	−16.72	−6.34	−9.65	−17.49	14:56:56	14:54:14	14:49:31	14:42:04	0:00:00	00:00:00	00:00:00	00:00:00
**Idoxuridine**	**0.84**	**143.7**	**63.0**	**30.6**	**36.8**	**260.81**	**58.10**	**−23.27**	**−7.53**	**N.D.**	**24:54:36**	**17:41:52**	**21:34:08**	**N.D.**	**00:00:00**	**00:00:00**	**00:00:00**
Lamivudine	0.77	31.69	32.69	34.67	37.03	−11.23	−8.43	−2.88	3.73	32:49:09	33:52:25	35:28:41	39:19:13	00:00:00	00:00:00	00:00:00	3:00:28
Maribavir	0.77	45.42	32.99	34.21	33.80	27.23	−7.58	−4.16	−5.32	46:44:06	35:09:24	33:29:27	35:01:33	10:25:21	00:00:00	00:00:00	00:00:00
6-mercaptopurine	0.77	27.72	28.97	37.02	39.53	−22.34	−18.85	3.70	10.72	34:55:04	32:30:01	37:22:22	38:19:02	00:00:00	00:00:00	1:03:37	2:00:17
Nelarabine	0.59	40.34	30.63	32.33	33.26	0.61	−23.61	−19.37	−17.05	48:25:49	34:32:22	35:15:53	34:33:34	4:36:33	00:00:00	00:00:00	00:00:00
Penciclovir	0.77	42.37	30.15	33.23	36.54	18.68	−15.56	−6.93	2.36	42:42:25	31:18:42	33:58:27	35:15:23	6:23:40	00:00:00	00:00:00	00:00:00
Pravastatin	0.59	30.29	37.85	36.52	45.23	−24.47	−5.61	−8.93	12.80	32:36:40	41:38:45	42:04:23	50:06:31	00:00:00	00:00:00	00:00:00	6:17:15
Proguanil hydrochloride	0.59	59.56	38.33	36.10	34.82	48.54	−4.42	−9.97	−13.18	65:51:23	46:29:08	40:48:49	36:43:29	22:02:07	2:39:52	00:00:00	00:00:00
Simvastatin	0.59	20.58	24.99	32.56	34.65	−48.67	−37.68	−18.80	−13.59	17:42:39	27:13:56	37:30:31	38:24:17	00:00:00	00:00:00	00:00:00	00:00:00
Sofosbuvir	0.59	35.76	34.01	33.28	35.31	−10.83	−15.19	−17.01	−11.95	41:47:34	39:39:17	33:18:38	37:03:26	00:00:00	00:00:00	00:00:00	00:00:00
Stavudine	0.77	30.20	32.23	32.94	37.85	−15.40	−9.71	−7.74	6.03	30:37:40	34:01:51	34:08:40	39:18:24	00:00:00	00:00:00	00:00:00	2:59:39
Telbivudine	0.59	39.04	31.31	35.43	30.51	−2.66	−21.92	−11.66	−23.91	47:38:45	37:15:51	38:54:50	35:46:09	3:49:29	00:00:00	00:00:00	00:00:00
Tenofovir disoproxil	0.77	52.88	93.87	43.49	34.89	48.14	162.94	21.81	−2.26	53:28:43	55:11:55	42:54:28	38:11:54	17:09:58	18:53:10	6:35:43	1:53:09
6-thioguanine	0.77	29.32	34.04	38.05	37.89	−17.86	−4.64	6.57	6.13	31:07:08	35:54:30	37:29:47	36:02:10	00:00:00	00:00:00	1:11:02	00:00:00
Valaciclovir	0.77	42.38	27.99	28.12	33.27	18.70	−21.59	−21.24	−6.81	43:09:03	28:31:33	30:35:19	33:07:52	6:50:18	00:00:00	00:00:00	00:00:00
**Valganciclovir hydrochloride**	**0.77**	**145.98**	**75.80**	**42.41**	**33.79**	**308.91**	**112.33**	**18.80**	**−5.35**	**N.D.**	**47:51:36**	**37:06:07**	**33:30:46**	**N.D.**	**11:32:51**	**0:47:22**	**00:00:00**
**Vidarabine**	**0.84**	**71.5**	**48.7**	**35.7**	**41.3**	**79.44**	**22.35**	**−10.34**	**3.77**	**70:50:12**	**48:46:26**	**19:17:51**	**26:50:37**	**40:54:08**	**18:50:22**	**00:00:00**	**00:00:00**

**Table 2:** List of the eight compounds presenting antiviral activity against the EHV-4 405/76 reference strain in vitro on E. Derm cells. Presented data are the mean ± standard deviation (S.D.) of three independent experiments. EC_50_: half-maximal effective concentration measured by impedance using RTCA and qPCR assay. CC_50_: half-maximal cytotoxic concentration measured by impedance using RTCA and CellTiter-Glo; >50 means that the compound did not show cytotoxicity at the highest concentration tested (50 µM). SI (Selectivity Index): ratio of CC_50_ obtained by RTCA and CellTiter-Glo to EC_50_ obtained by RTCA or by qPCR.

	RTCA				qPCR		CellTiter Glo	
	EC_50_ (µM ± S.D)	EC_50_ (µg/mL ± S.D)	CC_50_ (µM)	Si	EC_50_ (µM ± S.D)	EC_50_ (µg/mL ± S.D)	CC_50_ (µM)	SI
Aphidicolin (APD)	2.16 ± 0.18	0.73 ± 0 06	>50	>23.15	0.31 ± 0.09	0.11 ± 0.03	>50	>161.29
Cidofovir (CID)	12.55 ± 4.32	3.50 ± 1.21	>50	>3.98	7.99 ± 3.38	2.23 ± 0.94	>50	>6.26
Decitabine (DTB)	1.16 ± 0.31	0.26 ± 0.07	>50	>43.10	0.28 ± 0.05	0.06 ± 0.01	>50	>178.57
Ganciclovir (GCV)	4.05 ± 0.22	1.03 ± 0.06	>50	>12.35	1.32 ± 1.06	0.34 ± 0.27	>50	>37.88
Idoxuridine (IDX)	33.75 ± 15.85	11.95 ± 5.61	>50	>1.48	7.49 ± 1.00	2.65 ± 0.35	>50	>6.68
Pritelivir (BAY)	17.77 ± 1.20	7.15 ± 0.48	>50	>2.81	3.31 ± 0.89	1.33 ± 0.36	>50	>15.11
Valganciclovir (VGCV)	9.55 ± 2.81	3.38 ± 1.00	>50	>5.24	3.60 ± 1.02	1.28 ± 0.36	>50	>13.89
Vidarabine (VIR)	16.99 ± 4.50	4.85 ± 1.28	>50	>2.94	13.72 ± 1.92	3.91 ± 0.55	>50	>6.68

## Data Availability

The RNASeq gene expression data and raw fastq files are availablein the GEO repository (www.ncbi.nlm.nih.gov/geo/, e.g., accessed on 19 March 2024) under accession number: GSE261894.

## Supplementary Materials

The following supporting information can be downloaded at: https://www.mdpi.com/article/10.3390/v16050746/s1, Table S1: Library of selected compounds for the screening of antiviral effects by real-time cellular analysis against EHV-4 in vitro.

## References

[1] ICTV Current ICTV Taxonomy Release. https://ictv.global/taxonomy.

[2] Sabine M., Robertson G.R., Whalley J.M. (1981). Differentiation of Sub-Types of Equine Herpesvirus I by Restriction Endonuclease Analysis. Aust. Vet. J..

[3] Studdert M.J., Simpson T., Roizman B. (1981). Differentiation of Respiratory and Abortigenic Isolates of Equine Herpesvirus 1 by Restriction Endonucleases. Science.

[4] Telford E.A.R., Watson M.S., Perry J., Cullinane A.A., Davison A.J. (1998). The DNA Sequence of Equine Herpesvirus-4. DNA Seq..

[5] Pusterla N., James K., Barnum S., Bain F., Barnett D.C., Chappell D., Gaughan E., Craig B., Schneider C., Vaala W. (2022). Frequency of Detection and Prevalence Factors Associated with Common Respiratory Pathogens in Equids with Acute Onset of Fever and/or Respiratory Signs (2008–2021). Pathogens.

[6] O’Keefe J., Alley M., Jones D., Wilks C. (1995). Neonatal Mortality Due to Equid Herpesvirus 4 (EHV-4) in a Foal. Aust. Vet. J..

[7] Meyer H., Thein P., Hübert P. (1987). Characterization of Two Equine Herpesvirus (EHV) Isolates Associated with Neurological Disorders in Horses. Zentralblatt Vet. Reihe B J. Vet. Med. Ser. B.

[8] Borchers K., Wolfinger U., Ludwig H. (1999). Latency-Associated Transcripts of Equine Herpesvirus Type 4 in Trigeminal Ganglia of Naturally Infected Horses. J. Gen. Virol..

[9] Allen G.P., Kydd J.H., Slater J.D., Smith K.L. (2004). Equid Herpesvirus 1 and Equid Herpesvirus 4 Infections. Infectious Diseases of Livestock.

[10] Gilkerson J.R., Whalley J.M., Drummer H.E., Studdert M.J., Love D.N. (1999). Epidemiology of EHV-1 and EHV-4 in the Mare and Foal Populations on a Hunter Valley Stud Farm: Are Mares the Source of EHV-1 for Unweaned Foals. Vet. Microbiol..

[11] Ataseven V.S., Dağalp S.B., Güzel M., Başaran Z., Tan M.T., Geraghty B. (2009). Prevalence of Equine Herpesvirus-1 and Equine Herpesvirus-4 Infections in Equidae Species in Turkey as Determined by ELISA and Multiplex Nested PCR. Res. Vet. Sci..

[12] Ploszay G., Rola J., Larska M., Zmudzinski J.F. (2013). First Report on Equine Herpesvirus Type 4 Isolation in Poland--Evaluation of Diagnostic Tools. Pol. J. Vet. Sci..

[13] Badenhorst M., Page P., Ganswindt A., Laver P., Guthrie A., Schulman M. (2015). Detection of Equine Herpesvirus-4 and Physiological Stress Patterns in Young Thoroughbreds Consigned to a South African Auction Sale. BMC Vet. Res..

[14] Azab W., Bedair S., Abdelgawad A., Eschke K., Farag G.K., Abdel-Raheim A., Greenwood A.D., Osterrieder N., Ali A.A.H. (2019). Detection of Equid Herpesviruses among Different Arabian Horse Populations in Egypt. Vet. Med. Sci..

[15] El Brini Z., Fassi Fihri O., Paillot R., Lotfi C., Amraoui F., El Ouadi H., Dehhaoui M., Colitti B., Alyakine H., Piro M. (2021). Seroprevalence of Equine Herpesvirus 1 (EHV-1) and Equine Herpesvirus 4 (EHV-4) in the Northern Moroccan Horse Populations. Animals.

[16] Welch H.M., Bridges C.G., Lyon A.M., Griffiths L., Edington N. (1992). Latent Equid Herpesviruses 1 and 4: Detection and Distinction Using the Polymerase Chain Reaction and Co-Cultivation from Lymphoid Tissues. J. Gen. Virol..

[17] Borchers K., Wolfinger U., Lawrenz B., Schellenbach A., Ludwig H. (1997). Equine Herpesvirus 4 DNA in Trigeminal Ganglia of Naturally Infected Horses Detected by Direct in Situ PCR. J. Gen. Virol..

[18] Taouji S., Collobert C., Gicquel B., Sailleau C., Brisseau N., Moussu C., Breuil M.F., Pronost S., Borchers K., Zientara S. (2002). Detection and Isolation of Equine Herpesviruses 1 and 4 from Horses in Normandy: An Autopsy Study of Tissue Distribution in Relation to Vaccination Status. J. Vet. Med. B Infect. Dis. Vet. Public Health.

[19] Pusterla N., Mapes S., David Wilson W. (2012). Prevalence of Latent Alpha-Herpesviruses in Thoroughbred Racing Horses. Vet. J..

[20] Heldens J.G., Kersten A.J., Weststrate M.W., van den Hoven R. (2001). Duration of Immunity Induced by an Adjuvanted and Inactivated Equine Influenza, Tetanus and Equine Herpesvirus 1 and 4 Combination Vaccine. Vet. Q..

[21] Heldens J.G., Hannant D., Cullinane A.A., Prendergast M.J., Mumford J.A., Nelly M., Kydd J.H., Weststrate M.W., van den Hoven R. (2001). Clinical and Virological Evaluation of the Efficacy of an Inactivated EHV1 and EHV4 Whole Virus Vaccine (Duvaxyn EHV1,4). Vaccination/Challenge Experiments in Foals and Pregnant Mares. Vaccine.

[22] Pavulraj S., Eschke K., Theisen J., Westhoff S., Reimers G., Andreotti S., Osterrieder N., Azab W. (2021). Equine Herpesvirus Type 4 (EHV-4) Outbreak in Germany: Virological, Serological, and Molecular Investigations. Pathogens.

[23] Couroucé A., Normand C., Tessier C., Pomares R., Thévenot J., Marcillaud-Pitel C., Legrand L., Pitel P.-H., Pronost S., Lupo C. (2023). Equine Herpesvirus-1 Outbreak During a Show-Jumping Competition: A Clinical and Epidemiological Study. J. Equine Vet. Sci..

[24] De Clercq E., Li G. (2016). Approved Antiviral Drugs over the Past 50 Years. Clin. Microbiol. Rev..

[25] Murray M.J., del Piero F., Jeffrey S.C., Davis M.S., Furr M.O., Dubovi E.J., Mayo J.A. (1998). Neonatal Equine Herpesvirus Type 1 Infection on a Thoroughbred Breeding Farm. J. Vet. Intern. Med..

[26] Friday P.A., Scarratt W.K., Elvinger F., Timoney P.J., Bonda A. (2000). Ataxia and Paresis with Equine Herpesvirus Type 1 Infection in a Herd of Riding School Horses. J. Vet. Intern. Med..

[27] Henninger R.W., Reed S.M., Saville W.J., Allen G.P., Hass G.F., Kohn C.W., Sofaly C. (2007). Outbreak of Neurologic Disease Caused by Equine Herpesvirus-1 at a University Equestrian Center. J. Vet. Intern. Med..

[28] Garré B., Gryspeerdt A., Croubels S., De Backer P., Nauwynck H. (2009). Evaluation of Orally Administered Valacyclovir in Experimentally EHV1-Infected Ponies. Vet. Microbiol..

[29] Maxwell L.K., Bentz B.G., Gilliam L.L., Ritchey J.W., Pusterla N., Eberle R., Holbrook T.C., McFarlane D., Rezabek G.B., Meinkoth J. (2017). Efficacy of the Early Administration of Valacyclovir Hydrochloride for the Treatment of Neuropathogenic Equine Herpesvirus Type-1 Infection in Horses. Am. J. Vet. Res..

[30] Thieulent C.J., Sutton G., Toquet M.-P., Fremaux S., Hue E., Fortier C., Pléau A., Deslis A., Abrioux S., Guitton E. (2022). Oral Administration of Valganciclovir Reduces Clinical Signs, Virus Shedding and Cell-Associated Viremia in Ponies Experimentally Infected with the Equid Herpesvirus-1 C2254 Variant. Pathogens.

[31] Spiesschaert B., Osterrieder N., Azab W. (2015). Comparative Analysis of Glycoprotein B (gB) of Equine Herpesvirus Type 1 and Type 4 (EHV-1 and EHV-4) in Cellular Tropism and Cell-to-Cell Transmission. Viruses.

[32] Thieulent C.J., Hue E.S., Fortier C.I., Dallemagne P., Zientara S., Munier-Lehmann H., Hans A., Fortier G.D., Pitel P.-H., Vidalain P.-O. (2019). Screening and Evaluation of Antiviral Compounds against Equid Alpha-Herpesviruses Using an Impedance-Based Cellular Assay. Virology.

[33] Thieulent C., Hue E.S., Sutton G., Fortier C., Dallemagne P., Zientara S., Munier-Lehmann H., Hans A., Paillot R., Vidalain P.-O. (2020). Identification of Antiviral Compounds against Equid Herpesvirus-1 Using Real-Time Cell Assay Screening: Efficacy of Decitabine and Valganciclovir Alone or in Combination. Antiviral Res..

[34] Thieulent C., Fortier C., Munier-Lehmann H., Suzanne P., Dallemagne P., Zientara S., Hans A., Paillot R., Vidalain P.-O., Pronost S. (2020). Screening of Potential Antiviral Molecules against Equid Herpesvirus-1 Using Cellular Impedance Measurement: Dataset of 2,891 Compounds. Data Brief.

[35] Zhang J.H., Chung T.D., Oldenburg K.R. (1999). A Simple Statistical Parameter for Use in Evaluation and Validation of High Throughput Screening Assays. J. Biomol. Screen..

[36] Pan T., Huang B., Zhang W., Gabos S., Huang D.Y., Devendran V. (2013). Cytotoxicity Assessment Based on the AUC50 Using Multi-Concentration Time-Dependent Cellular Response Curves. Anal. Chim. Acta.

[37] Diallo I.S., Hewitson G., Wright L.L., Kelly M.A., Rodwell B.J., Corney B.G. (2007). Multiplex Real-Time PCR for the Detection and Differentiation of Equid Herpesvirus 1 (EHV-1) and Equid Herpesvirus 4 (EHV-4). Vet. Microbiol..

[38] Jourdren L., Bernard M., Dillies M.-A., Le Crom S. (2012). Eoulsan: A Cloud Computing-Based Framework Facilitating High Throughput Sequencing Analyses. Bioinformatics.

[39] Dobin A., Davis C.A., Schlesinger F., Drenkow J., Zaleski C., Jha S., Batut P., Chaisson M., Gingeras T.R. (2013). STAR: Ultrafast Universal RNA-Seq Aligner. Bioinformatics.

[40] Li H., Handsaker B., Wysoker A., Fennell T., Ruan J., Homer N., Marth G., Abecasis G., Durbin R., 1000 Genome Project Data Processing Subgroup (2009). The Sequence Alignment/Map Format and SAMtools. Bioinformatics.

[41] Anders S., Pyl P.T., Huber W. (2015). HTSeq—A Python Framework to Work with High-Throughput Sequencing Data. Bioinformatics.

[42] Love M.I., Huber W., Anders S. (2014). Moderated Estimation of Fold Change and Dispersion for RNA-Seq Data with DESeq2. Genome Biol..

[43] Huang D.W., Sherman B.T., Lempicki R.A. (2009). Systematic and Integrative Analysis of Large Gene Lists Using DAVID Bioinformatics Resources. Nat. Protoc..

[44] Sherman B.T., Hao M., Qiu J., Jiao X., Baseler M.W., Lane H.C., Imamichi T., Chang W. (2022). DAVID: A Web Server for Functional Enrichment Analysis and Functional Annotation of Gene Lists (2021 Update). Nucleic Acids Res..

[45] Kagan A.B., Garrison D.A., Anders N.M., Webster J.A., Baker S.D., Yegnasubramanian S., Rudek M.A. (2023). DNA Methyltransferase Inhibitor Exposure-Response: Challenges and Opportunities. Clin. Transl. Sci..

[46] Betz U.A.K., Fischer R., Kleymann G., Hendrix M., Rübsamen-Waigmann H. (2002). Potent in Vivo Antiviral Activity of the Herpes Simplex Virus Primase-Helicase Inhibitor BAY 57-1293. Antimicrob. Agents Chemother..

[47] Azab W., Tsujimura K., Kato K., Arii J., Morimoto T., Kawaguchi Y., Tohya Y., Matsumura T., Akashi H. (2010). Characterization of a Thymidine Kinase-Deficient Mutant of Equine Herpesvirus 4 and in Vitro Susceptibility of the Virus to Antiviral Agents. Antiviral Res..

[48] Zhang J., Yang G., Wang X., Zhu Y., Wang J. (2022). 25-Hydroxycholesterol Mediates Cholesterol Metabolism to Restrict Porcine Deltacoronavirus Infection via Suppression of Transforming Growth Factor Β1. Microbiol. Spectr..

[49] Españo E., Kim J.-K. (2022). Effects of Statin Combinations on Zika Virus Infection in Vero Cells. Pharmaceutics.

[50] Osuna-Ramos J.F., Farfan-Morales C.N., Cordero-Rivera C.D., De Jesús-González L.A., Reyes-Ruiz J.M., Hurtado-Monzón A.M., Palacios-Rápalo S.N., Jiménez-Camacho R., Meraz-Ríos M.A., Del Ángel R.M. (2023). Cholesterol-Lowering Drugs as Potential Antivirals: A Repurposing Approach against Flavivirus Infections. Viruses.

[51] Kleymann G. (2004). Helicase Primase: Targeting the Achilles Heel of Herpes Simplex Viruses. Antivir. Chem. Chemother..

[52] Biswas S., Kleymann G., Swift M., Tiley L.S., Lyall J., Aguirre-Hernández J., Field H.J. (2008). A Single Drug-Resistance Mutation in HSV-1 UL52 Primase Points to a Difference between Two Helicase-Primase Inhibitors in Their Mode of Interaction with the Antiviral Target. J. Antimicrob. Chemother..

[53] Vissani M.A., Zabal O., Tordoya M.S., Parreño V., Thiry E., Barrandeguy M. (2018). In Vitro Comparison of Acyclovir, Ganciclovir and Cidofovir against Equid Alphaherpesvirus 3 and Evaluation of Their Efficacy against Six Field Isolates. Rev. Argent. Microbiol..

[54] Smith K.O., Galloway K.S., Hodges S.L., Ogilvie K.K., Radatus B.K., Kalter S.S., Heberling R.L. (1983). Sensitivity of Equine Herpesviruses 1 and 3 in Vitro to a New Nucleoside Analogue, 9-[[2-Hydroxy-1-(Hydroxymethyl) Ethoxy] Methyl] Guanine. Am. J. Vet. Res..

[55] Garré B., van der Meulen K., Nugent J., Neyts J., Croubels S., De Backer P., Nauwynck H. (2007). In Vitro Susceptibility of Six Isolates of Equine Herpesvirus 1 to Acyclovir, Ganciclovir, Cidofovir, Adefovir, PMEDAP and Foscarnet. Vet. Microbiol..

[56] Carmichael R.J., Whitfield C., Maxwell L.K. (2013). Pharmacokinetics of Ganciclovir and Valganciclovir in the Adult Horse. J. Vet. Pharmacol. Ther..

[57] Piret J., Goyette N., Boivin G. (2016). Novel Method Based on Real-Time Cell Analysis for Drug Susceptibility Testing of Herpes Simplex Virus and Human Cytomegalovirus. J. Clin. Microbiol..

[58] Ledbetter E.C., Nicklin A.M., Spertus C.B., Pennington M.R., Van de Walle G.R., Mohammed H.O. (2018). Evaluation of Topical Ophthalmic Ganciclovir Gel for the Treatment of Dogs with Experimentally Induced Ocular Canine Herpesvirus-1 Infection. Am. J. Vet. Res..

[59] Goodman L.B., Loregian A., Perkins G.A., Nugent J., Buckles E.L., Mercorelli B., Kydd J.H., Palù G., Smith K.C., Osterrieder N. (2007). A Point Mutation in a Herpesvirus Polymerase Determines Neuropathogenicity. PLoS Pathog..

[60] Sutton G., Thieulent C., Fortier C., Hue E.S., Marcillaud-Pitel C., Pléau A., Deslis A., Guitton E., Paillot R., Pronost S. (2020). Identification of a New Equid Herpesvirus 1 DNA Polymerase (ORF30) Genotype with the Isolation of a C2254/H752 Strain in French Horses Showing No Major Impact on the Strain Behaviour. Viruses.

[61] Sheaff R., Ilsley D., Kuchta R. (1991). Mechanism of DNA Polymerase Alpha Inhibition by Aphidicolin. Biochemistry.

[62] Xiao J., Liu P., Wang Y., Zhu Y., Zeng Q., Hu X., Ren Z., Wang Y. (2022). A Novel Cognition of Decitabine: Insights into Immunomodulation and Antiviral Effects. Molecules.

[63] Colwell M., Wanner N.M., Drown C., Drown M., Dolinoy D.C., Faulk C. (2021). Paradoxical Whole Genome DNA Methylation Dynamics of 5′aza-Deoxycytidine in Chronic Low-Dose Exposure in Mice. Epigenetics.

[64] Birdwell C.E., Queen K.J., Kilgore P.C.S.R., Rollyson P., Trutschl M., Cvek U., Scott R.S. (2014). Genome-Wide DNA Methylation as an Epigenetic Consequence of Epstein-Barr Virus Infection of Immortalized Keratinocytes. J. Virol..

[65] Chiappinelli K.B., Strissel P.L., Desrichard A., Li H., Henke C., Akman B., Hein A., Rote N.S., Cope L.M., Snyder A. (2015). Inhibiting DNA Methylation Causes an Interferon Response in Cancer via dsRNA Including Endogenous Retroviruses. Cell.

[66] Roulois D., Loo Yau H., Singhania R., Wang Y., Danesh A., Shen S.Y., Han H., Liang G., Jones P.A., Pugh T.J. (2015). DNA-Demethylating Agents Target Colorectal Cancer Cells by Inducing Viral Mimicry by Endogenous Transcripts. Cell.

[67] Cayley P.J., Davies J.A., McCullagh K.G., Kerr I.M. (1984). Activation of the Ppp(A2′p)nA System in Interferon-Treated, Herpes Simplex Virus-Infected Cells and Evidence for Novel Inhibitors of the Ppp(A2′p)nA-Dependent RNase. Eur. J. Biochem..

[68] Glorieux S., Vandekerckhove A.P., Goris N., Yang X.-Y., Steukers L., Van de Walle G.R., Croubels S., Neyts J., Nauwynck H.J. (2012). Evaluation of the Antiviral Activity of (1′S,2′R)-9-[[1′,2′-Bis(Hydroxymethyl)Cycloprop-1′-Yl]Methyl]Guanine (A-5021) against Equine Herpesvirus Type 1 in Cell Monolayers and Equine Nasal Mucosal Explants. Antiviral Res..

[69] Rybak-Wolf A., Wyler E., Pentimalli T.M., Legnini I., Oliveras Martinez A., Glažar P., Loewa A., Kim S.J., Kaufer B.B., Woehler A. (2023). Modelling Viral Encephalitis Caused by Herpes Simplex Virus 1 Infection in Cerebral Organoids. Nat. Microbiol..

[70] Nagy K., Sung H.-K., Zhang P., Laflamme S., Vincent P., Agha-Mohammadi S., Woltjen K., Monetti C., Michael I.P., Smith L.C. (2011). Induced Pluripotent Stem Cell Lines Derived from Equine Fibroblasts. Stem Cell Rev. Rep..

[71] Breton A., Sharma R., Diaz A.C., Parham A.G., Graham A., Neil C., Whitelaw C.B., Milne E., Donadeu F.X. (2013). Derivation and Characterization of Induced Pluripotent Stem Cells from Equine Fibroblasts. Stem Cells Dev..

[72] Fortuna P.R.J., Bielefeldt-Ohmann H., Ovchinnikov D.A., Wolvetang E.J., Whitworth D.J. (2018). Cortical Neurons Derived from Equine Induced Pluripotent Stem Cells Are Susceptible to Neurotropic Flavivirus Infection and Replication: An In Vitro Model for Equine Neuropathic Diseases. Stem Cells Dev..

