# Supplementary material for: A Screening Study Identified Decitabine as an Inhibitor of Equid Herpesvirus 4 That Enhances the Innate Antiviral Response
Source: Viruses. 2024 May 8;16(5):746. doi: 10.3390/v16050746 (PMC11125953; doi:10.3390/v16050746)
Supplement: Supplementary file 1 [file viruses-16-00746-s001.zip › viruses-2961355-supplementary.pdf]

Supplementary Table S1: Library of selected compounds for the screening and dose-response assay of antiviral effect by real-time cellular analysis against EHV-4 *in vitro*. N.K.: Not Know

Compound name	Supplier	Catalogue no.	Application	Stock solution in DMSO (mM)
2'-C-methylcytidine	Ark Phar, Inc.	AK-88474	Screening	10
25-hydroxycholesterol	Cayman Europe	11097	Screening	10
Abacavir sulfate	TargetMol	T6367	Screening	10
Aciclovir	VWR	ABCAAB120631-100	Screening	20
Adefovir dipivoxil	TargetMol	T1675	Screening	10
Aphidicolin	Santa Cruz Biotechnology	Sc-201535	Screening and dose-response assay	20
Arbidol (Umifenovir hydrochloride)	TargetMol	T0104	Screening	10
Atorvastatin	N.K.	N.K.	Screening	10
BAY 57-1293	MedChem express	HY-13303/CS-1693	Screening and dose-response assay	20
Brivudine	MedChem express	HY-13578/CS-6292	Screening	10
Capecitabine	TargetMol	T1408	Screening	10
Cidofovir	TargetMol	T6244	Screening and dose-response assay	10
Cytarabine	TargetMol	T1272	Screening	10
Decitabine	TargetMol	T1508	Screening	10
Decitabine	MedChem express	HY-A0004	Dose-response assay	20
Didanosine	TargetMol	T2578	Screening	10
DMXAA (Vadimezan)	TargetMol	T6273	Screening	10
Dynasore	MedChem express	HY-15304	Screening	20
Eflornithin (dfmo)	Sigma Aldrich (Merck)	D193	Screening	10
Emtricitabine	TargetMol	T6214	Screening	10
Famciclovir	TargetMol	T1646	Screening	10
Favipiravir	TargetMol	T6833	Screening	10
5-fluorouracil	AK Scientific, Inc.	C526	Screening	10

Fluvastatin	N.K.	N.K.	Screening	10
Ganciclovir	Clinisciences	A11645-50	Screening and dose-response assay	10
Genistein	Sigma Aldrich (Merck)	G6649-5mg	Screening	20
Idoxuridine	MedChem Tronica	HY-B0307	Screening and dose-response assay	20
Lamivudine	TargetMol	T0682	Screening	10
Maribavir	TargetMol	T2162	Screening	10
6-mercaptopurine	TargetMol	T0010	Screening	10
Nelarabine	AK Scientific, Inc.	R077	Screening	10
Penciclovir	TargetMol	T1643	Screening	10
Pravastatin	N.K.	N.K.	Screening	10
Proguanil hydrochloride	BIONET / Key Organics Ltd.	HS-0102	Screening	10
Simvastatin	N.K.	N.K.	Screening	10
Sofosbuvir	TargetMol	T6676	Screening	10
Stavudine	TargetMol	T1404	Screening	10
Telbivudine	TargetMol	T1513	Screening	10
Tenofovir alafenamide	TargetMol	T2409	Screening	10
6-thioguanine	TargetMol	T3089	Screening	10
Valaciclovir hydrochloride	TargetMol	T1087	Screening	10
Valganciclovir hydrochloride	TargetMol	T1533	Screening	10
Valganciclovir	MedChem express	HY-A0032A	Dose-response assay	20
Vidarabine	CliniSciences	HY-B0277-100mg	Screening and dose-response assay	10
